# Supplementary material for: A mediator of OsbZIP46 deactivation and degradation negatively regulates seed dormancy in rice
Source: Nat Commun. 2024 Feb 7;15:1134. doi: 10.1038/s41467-024-45402-z (PMC10850359; doi:10.1038/s41467-024-45402-z)
Supplement: Supplementary file 1 — Supplementary Information [file 41467_2024_45402_MOESM1_ESM.pdf]

A mediator of OsbZIP46 deactivation and degradation negatively regulates seed dormancy in rice

Guo *et al*.

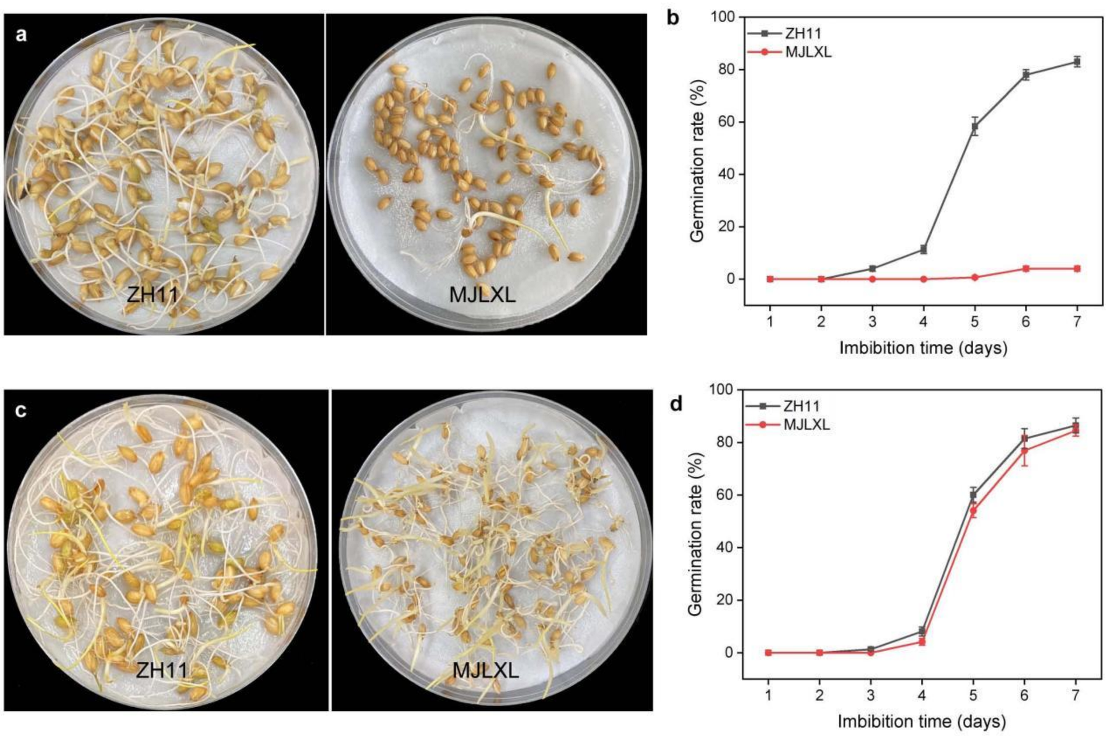

Supplementary Figure 1. Identification of parental phenotypes. a The 7-day germination phenotype of parents 35 DAH. **b** Time-course germination percentage of parents 35 DAH. **c** The 7-day germination phenotype of both parents three months after harvest. **d** Time-course germination percentage of parents three months after harvest. Data are presented as the mean ± SD, n = 3 independent experiments. Source data are provided as a Source Data file.

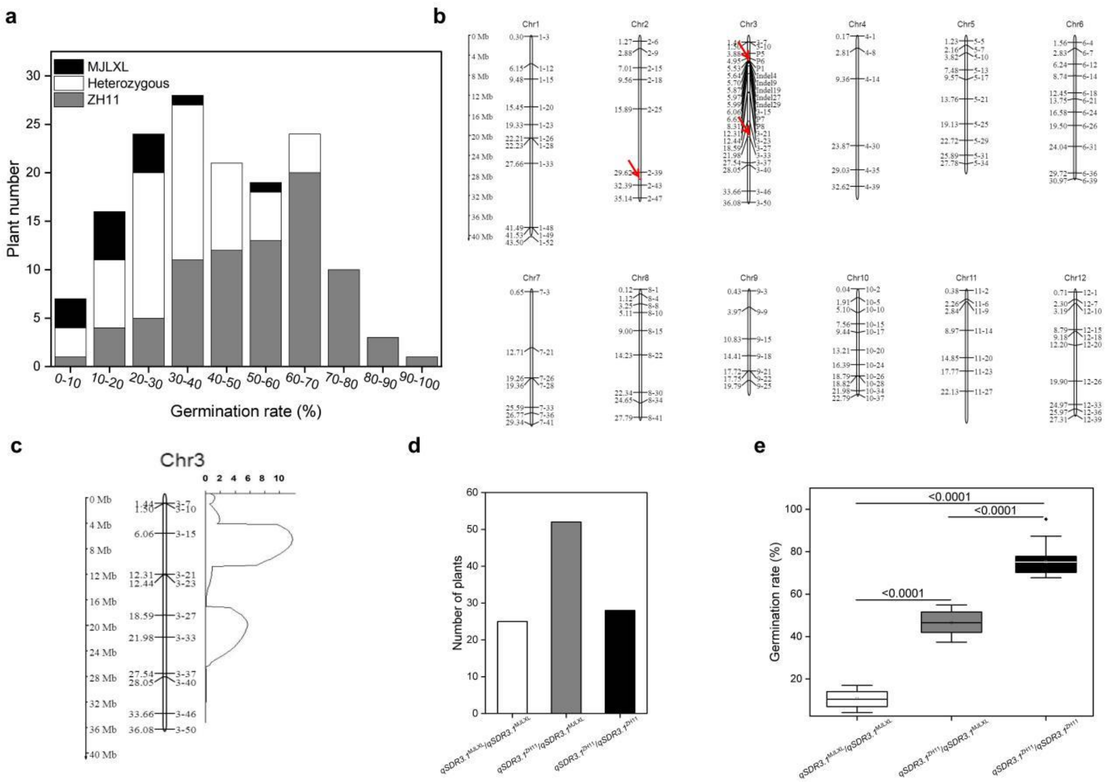

Supplementary Figure 2. Chromosomal locations of QTLs for seed dormancy. a Distribution of seed dormancy traits in the subpopulation of 153 plants. **b** There are three rice dormancy QTLs on chromosomes 2 and 3. The red triangle indicates the mapped seed dormancy QTLs. **c** The LOD on chromosome 3. The number on the left of the chromosome represents the physical distance (Mb) between two markers, and the number on the right of the chromosome represents the marker name. **d** Segregation of the BC_3F_2 population derived from ZH11 and MJLXL. **e** Box plots for germination rate among homozygotes for the MJLXL *qSDR3.1* allele, heterozygotes for the MJLXL *qSDR3.1* allele and the ZH11 *qSDR3.1* allele, and homozygotes for the ZH11 *qSDR3.1* allele. Data are presented as the mean ± SD, and *P* values are indicated by two-tailed Student's *t* test. n = 3 independent experiments. Source data are provided as a Source Data file.

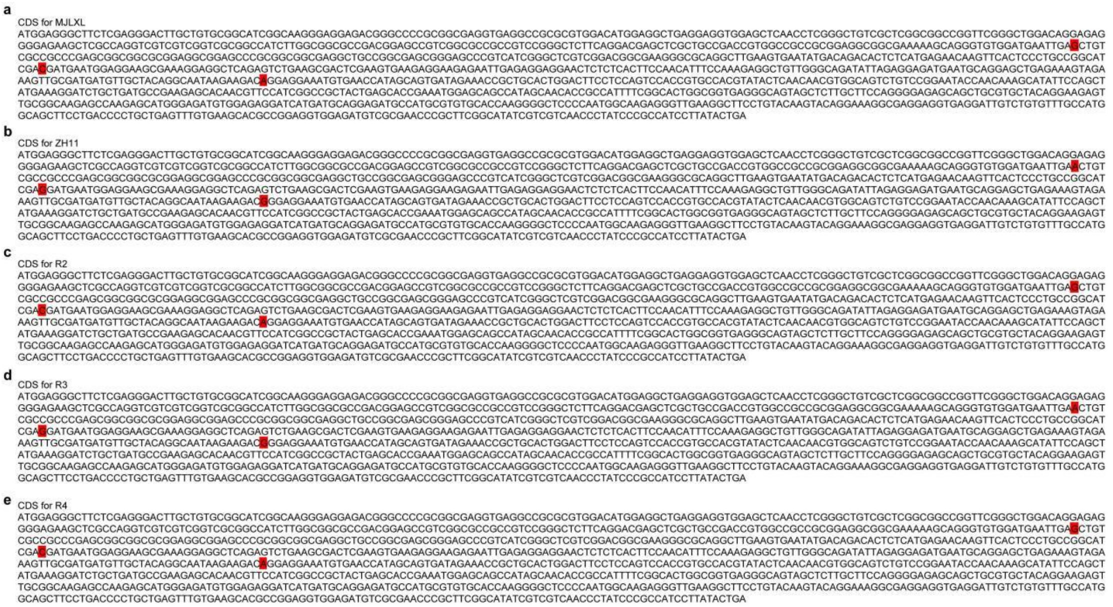

Supplementary Figure 3. cDNA sequences of the parents and Rs.

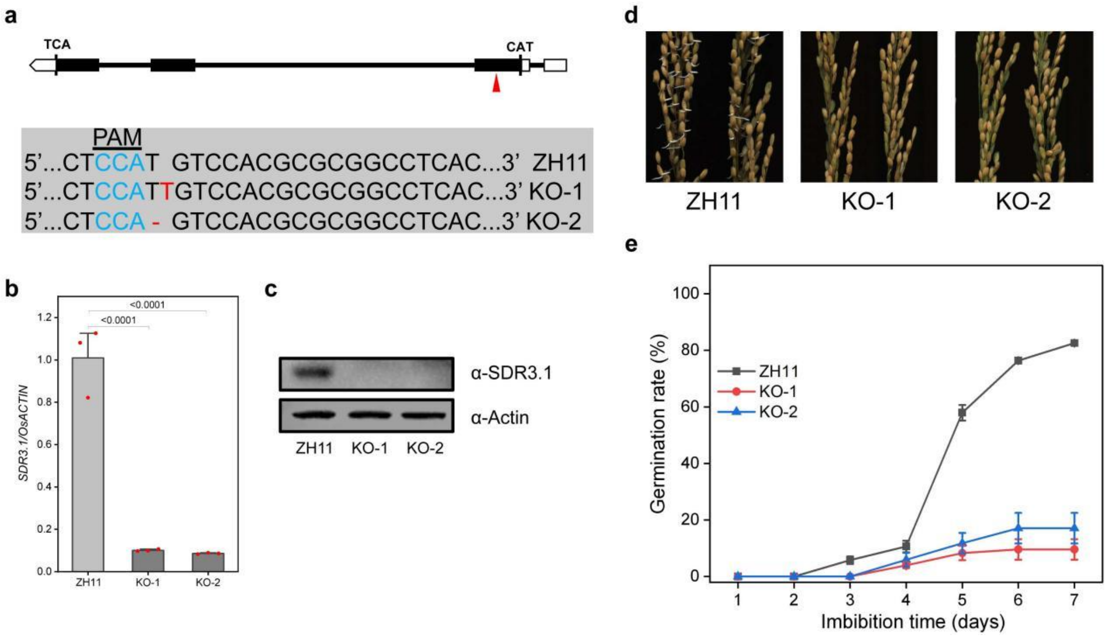

Supplementary Figure 4. Phenotype of *SDR3.1* knockout lines. a *SDR3.1* mutant sites of two knockout lines; red arrows indicates knockout sites. **b** Relative expression levels of *SDR3.1* in two knockout lines and wild-type ZH11. **c** Protein levels of *SDR3.1* in two knockout lines and wild-type ZH11. The experiments were replicated 3 times with similar results. **d** Germination performance of mature seeds of two knockout lines and wild-type ZH11 35 DAH. **e** Time-course germination percentage of mature seeds of two knockout lines and wild-type ZH11 35 DAH. Data are presented as the mean ± SD, and *P* values are indicated by two-tailed Student's *t* test. n = 3 independent experiments. Source data are provided as a Source Data file.

```
ZH11        MEGFSRDLLC GIGKGGDGPR GEVRPRVDME AEEVELNLGL SLGGRFGLDR
KO-1        MEGFSRDLLC GIGKGGDGPR GEVRPRVDNG G--------- ----------
KO-2        MEGFSRDLLC GIGKGGDGPR GEVRPRVDWR LRRWSSTSGC RSAAGSGWTG

            RG-EKLARSS SVAAILAAPT EPSAPPSGLF RTSSLPTVAA AEAAKKQGVD
            ---------- ---------- ---------- ---------- ----------
            EGRSSPGRRR SRPSWRRRRS RRRRRPGSSG RARCRPWPPR RRRKSRVWMN

            ELNCRRPSGG AEAEPAAARL PASGSPSSGS SDGEGRRLEV NMTDTLMRTS
            ---------- ---------- ---------- ---------- ----------
            ---------- ---------- ---------- ---------- ----------

            SLPAGIEDEW RKRKEAQSLK RLEVKRKRIE RRNSLTSNIS KEAVGQILEE
            ---------- ---------- ---------- ---------- ----------
            ---------- ---------- ---------- ---------- ----------

            MNAGAEKVES CDDVATGNKK TGGNVNHSSD RNRCTGLPPV HRATYTQQRG
            ---------- ---------- ---------- ---------- ----------
            ---------- ---------- ---------- ---------- ----------

            SLSGIPTKHI PAMKGSADAE EHNVPSAATE HRNGAAIATP PFSALAVRAV
            ---------- ---------- ---------- ---------- ----------
            ---------- ---------- ---------- ---------- ----------

            ALASRGEQLR ATGRVAARAK SMGDVERIMM QEMPCVCTKG LPNGKRVEGF
            ---------- ---------- ---------- ---------- ----------
            ---------- ---------- ---------- ---------- ----------

            LYKYRKGEEV RIVCVCHGSF LTPAEFVKHA GGGDVANPLR HIVVNPIPPS
            ---------- ---------- ---------- ---------- ----------
            ---------- ---------- ---------- ---------- ----------

            LY
            --
            --
```

Supplementary Figure 5. Protein sequence of *SDR3.1* in ZH11, KO-1, and KO-2.

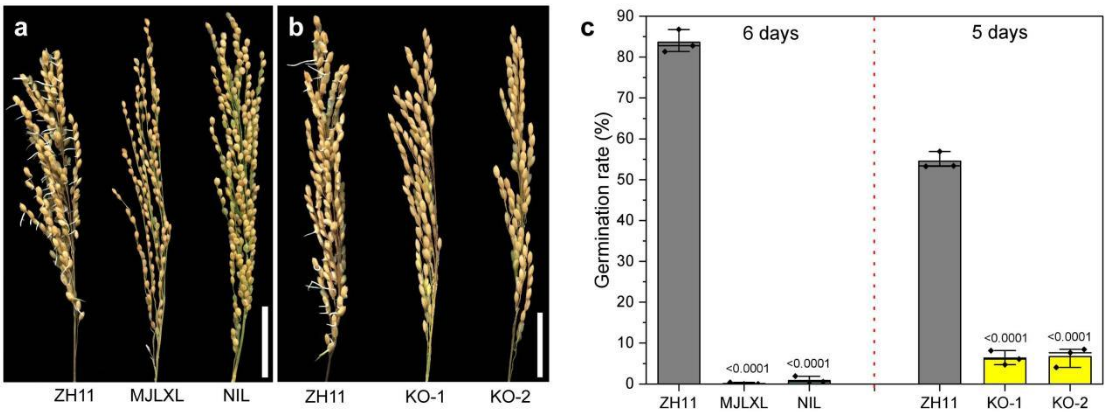

Supplementary Figure 6. PHS of different materials. PHS of ZH11, MJLXL and NIL (**a**) and ZH11, KO-1 and KO-2 (**b**). Bar = 10 cm. **c** Germination rate of ZH11, MJLXL and NIL at 6th day and of ZH11, KO-1 and KO-2 at 5th day. Data are presented as the mean ± SD, and *P* values are indicated by two-tailed Student's *t* test. n = 3 independent experiments. Source data are provided as a Source Data file.

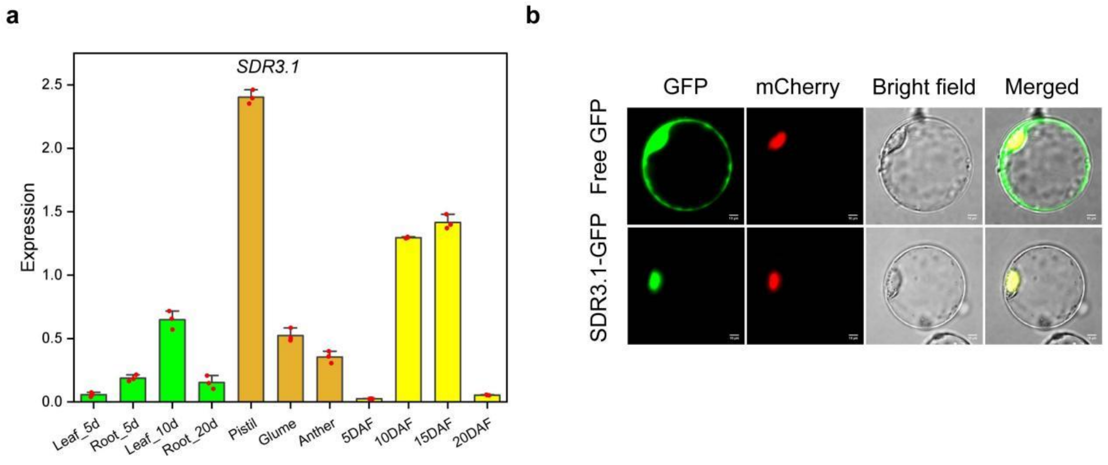

Supplementary Figure 7. Expression pattern and subcellular localization of *SDR3.1*. a Relative expression levels of *SDR3.1* in different tissues. Data are presented as the mean ± SD (n = 3 independent experiments). **b** Subcellular localization of *SDR3.1* in rice protoplasts. The signals from GFP fluorescence, mCherry, bright field, and merged images are shown. The experiments were replicated 3 times with similar results. Bar = 10 μm. Source data are provided as a Source Data file.

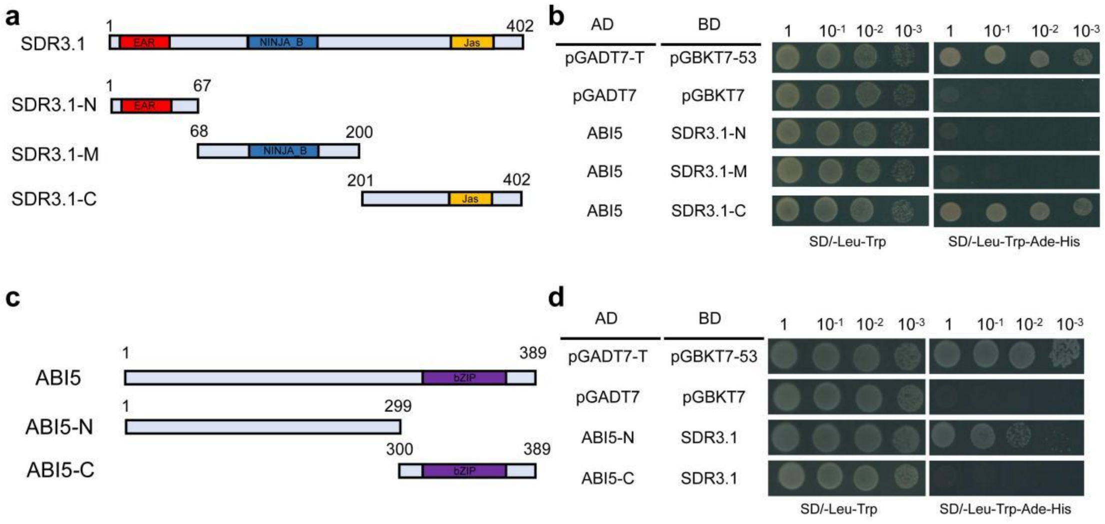

Supplementary Figure 8. Y2H screening assays were used to identify the regions of ABI5 interacted with SDR3.1. a Diagrams showing the conserved EAR domain, NINJA_B domain, and Jas domain of SDR3.1 and the truncated versions of SDR3.1 in the assays. SDR3.1-N represents amino acids 1-67; SDR3.1-M represents amino acids 68-200, and SDR3.1-C represents amino acids 201-402. **b** Y1H assay showed the interaction of truncated versions of SDR3.1 with the full-length ABI5 protein. **c** Diagrams showing the conserved bZIP domain of ABI5 and the truncated versions of ABI5 in the assays. ABI5-N represents amino acids 1-299 and ABI5-C represents amino acids 300-389. **d** Y2H assay showed the interaction of truncated versions of ABI5 with the full-length SDR3.1 protein.

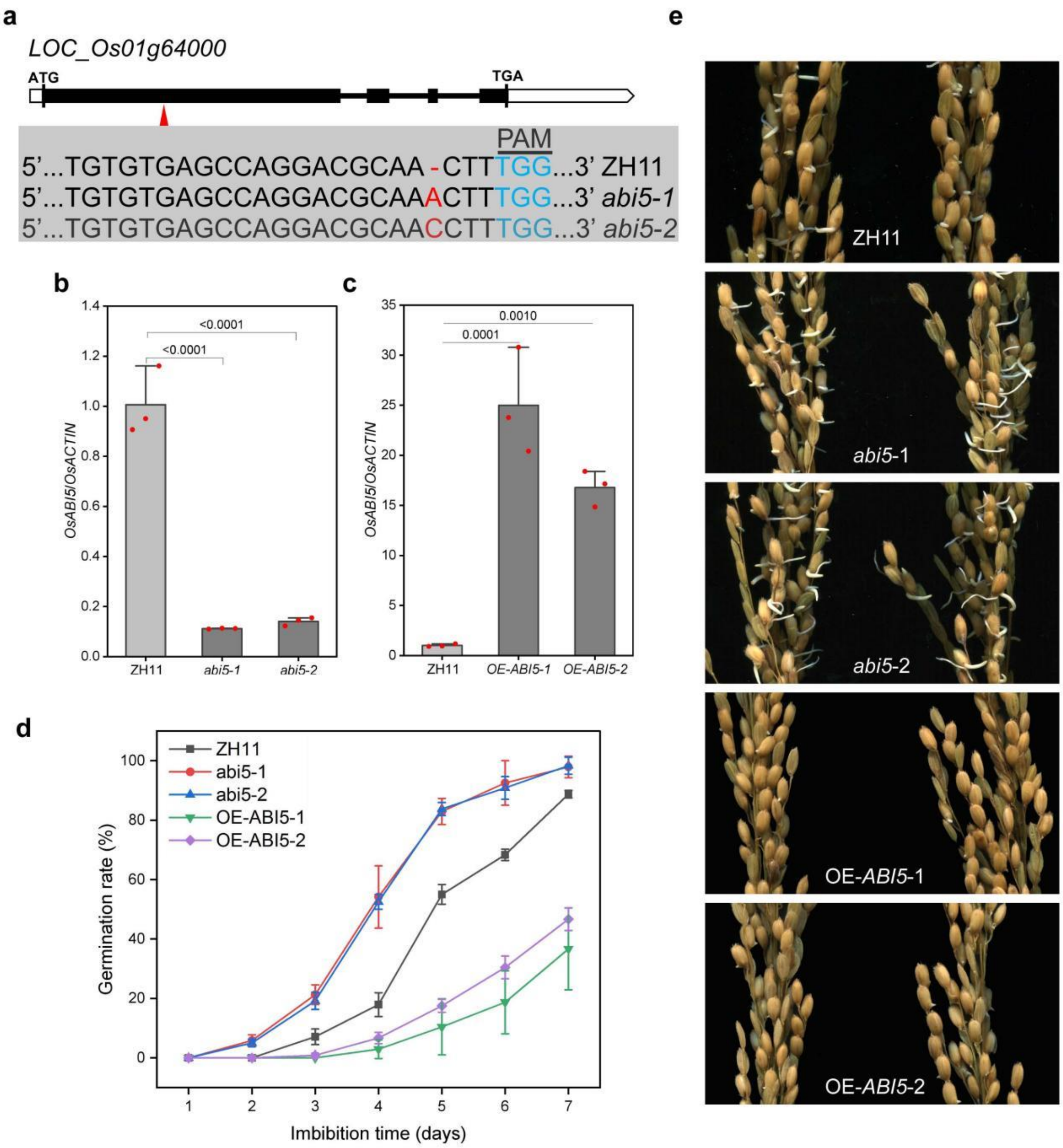

Supplementary Figure 9. Phenotypes of the *ABI5* knockout lines and OE lines. a *ABI5* mutation sites of two knockout lines; red arrows indicate knockout sites. **b** Relative expression levels of *ABI5* in two knockout lines and wild-type ZH11. **c** Relative expression levels of *ABI5* in two OE lines and wild-type ZH11. **d** Time-course germination percentage of two mutant lines, two OE lines and ZH11 35 DAH. **e** PHS phenotypes of two mutant lines, two OE lines and ZH11 35 DAH. Data are presented as the mean ± SD, and *P* values are indicated by two-tailed Student's *t* test. n = 3 independent experiments. Source data are provided as a Source Data file.

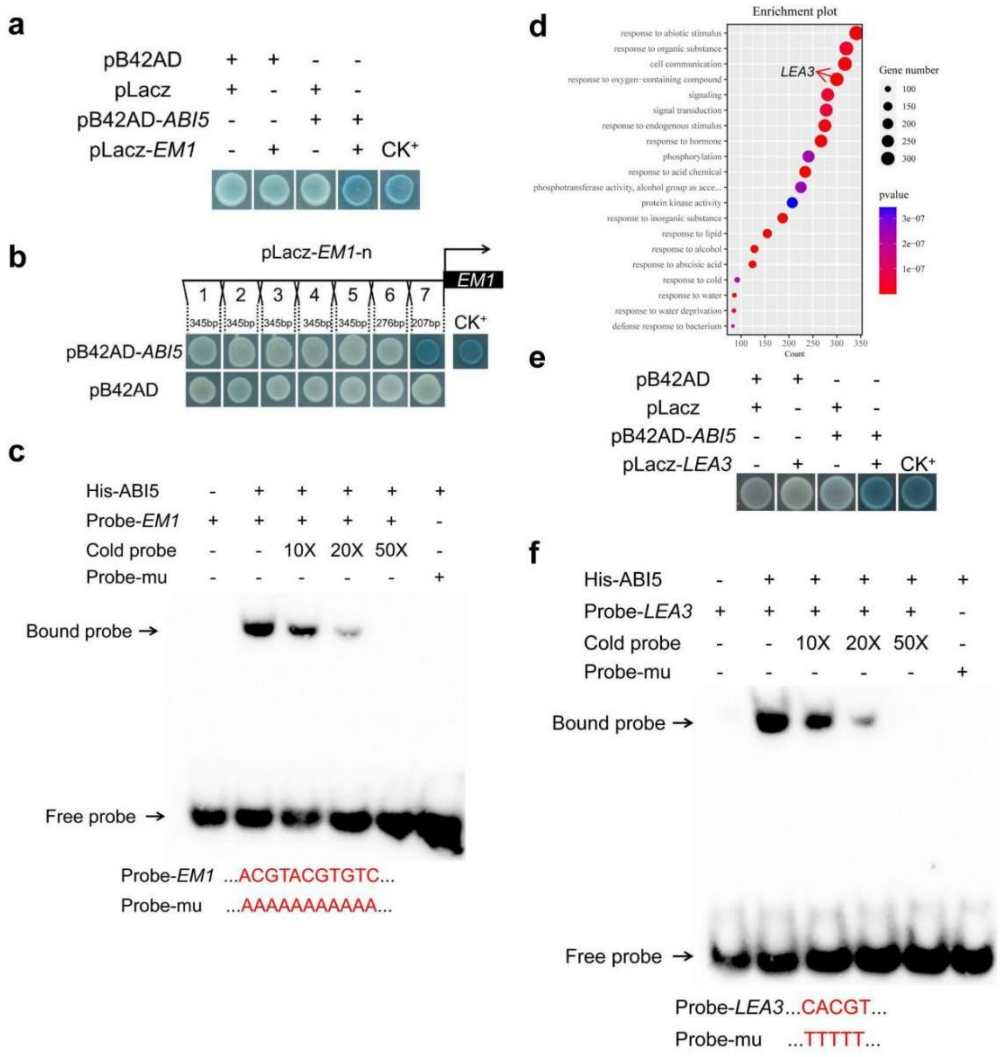

Supplementary Figure 10. *ABI5* bound to the promoters of *EM1* and *LEA3*. a Y1H assay showed that *ABI5* bound to the *EM1* promoter (2000 bp upstream of ATG). **b** Y1H assay showed that *ABI5* bound to the *EM1* promoter (207 bp upstream of ATG). **c** EMSA showed that *ABI5* bound to the biotin probe on the promoter of *EM1*. The experiments were replicated 3 times with similar results. **d** Cut&Tag of ABI5_go_enrich_result. **e** Y1H assay showed that ABI5 bound to the *LEA3* promoter (2000 bp upstream of ATG). **f** EMSA showed that ABI5 bound to the biotin probe on the promoter of *LEA3*. The experiments were replicated 3 times with similar results. Source data are provided as a Source Data file.

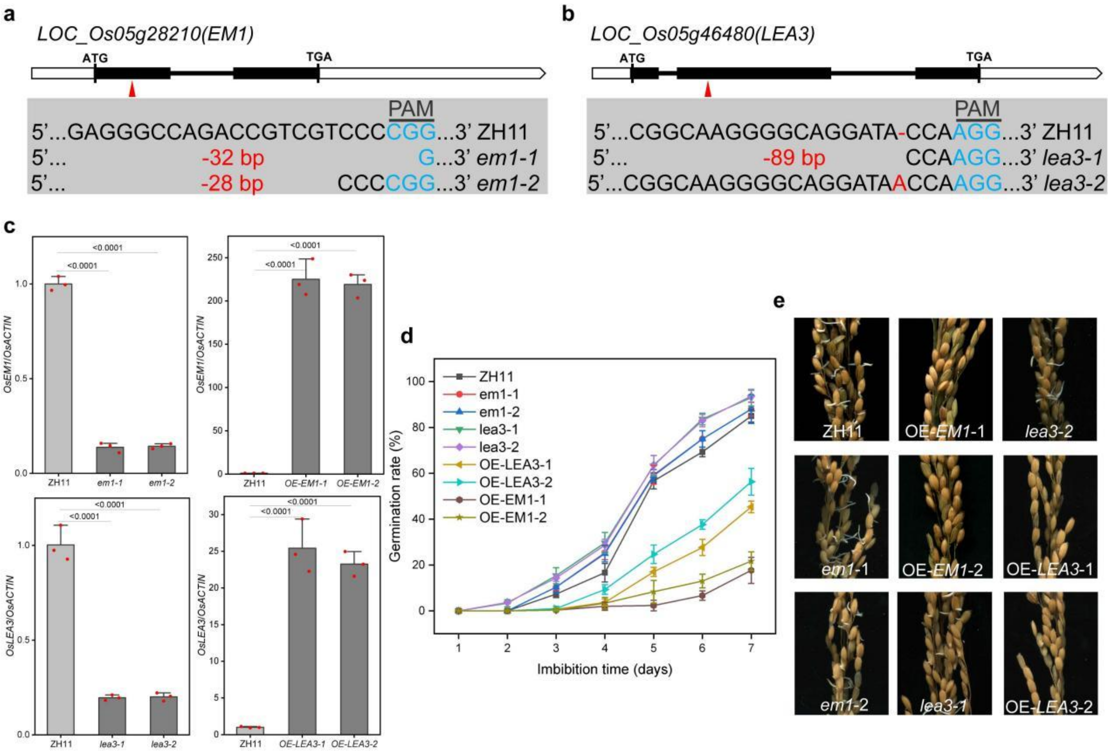

Supplementary Figure 11. Phenotypes of the *EM1* and *LEA3* knockout lines and OE lines. a *EM1* mutation sites of two knockout lines; red arrows indicate knockout sites. **b** *LEA3* mutation sites of two knockout lines; red arrows indicate knockout sites. **c** Relative expression levels of *EM1* or *LEA3* in two knockout lines and two OE lines. **d** Time-course germination percentage of four mutant lines, four OE lines and ZH11 35 DAH. **e** PHS phenotypes of four mutant lines, four OE lines and ZH11 35 DAH. Data are presented as the mean ± SD, and P values are indicated by two-tailed Student's t test. n = 3 independent experiments. Source data are provided as a Source Data file.

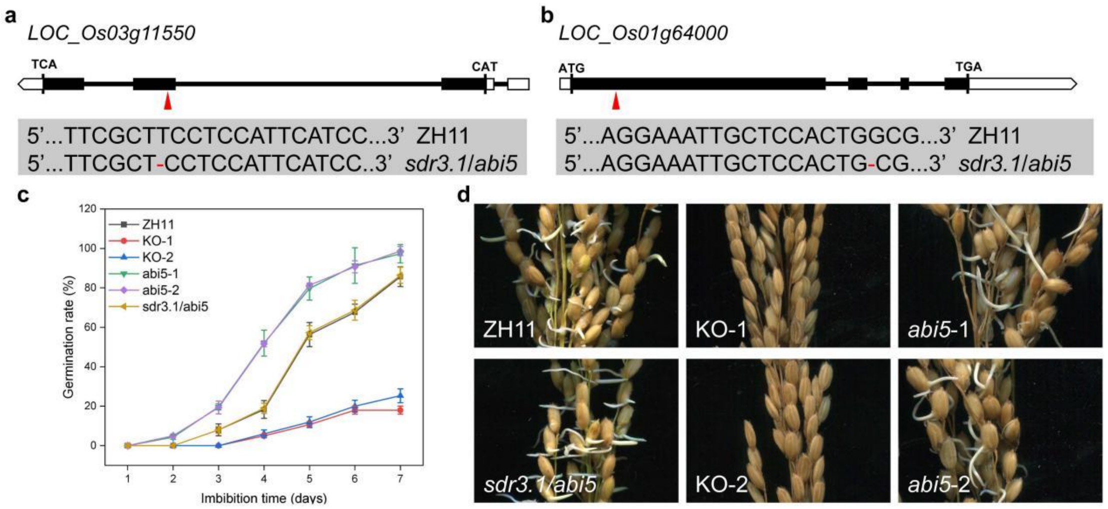

Supplementary Figure 12. Phenotype of the *SDR3.1/ABI5* double knockout line. a *SDR3.1* mutation site of *SDR3.1/ABI5* double knockout line; red arrows indicate knockout sites. **b** *ABI5* mutation site of *SDR3.1/ABI5* double knockout line; red arrows indicate knockout sites. **c** Time-course germination percentage of KO-1, KO-2, *abi5-1*, *abi5-2*, *SDR3.1/ABI5* double knockout line and ZH11 35 DAH. **d** PHS phenotypes of KO-1, KO-2, *abi5-1*, *abi5-2*, *SDR3.1/ABI5* double knockout line and ZH11 35 DAH. Data are presented as the mean ± SD. n = 3 independent experiments. Source data are provided as a Source Data file.

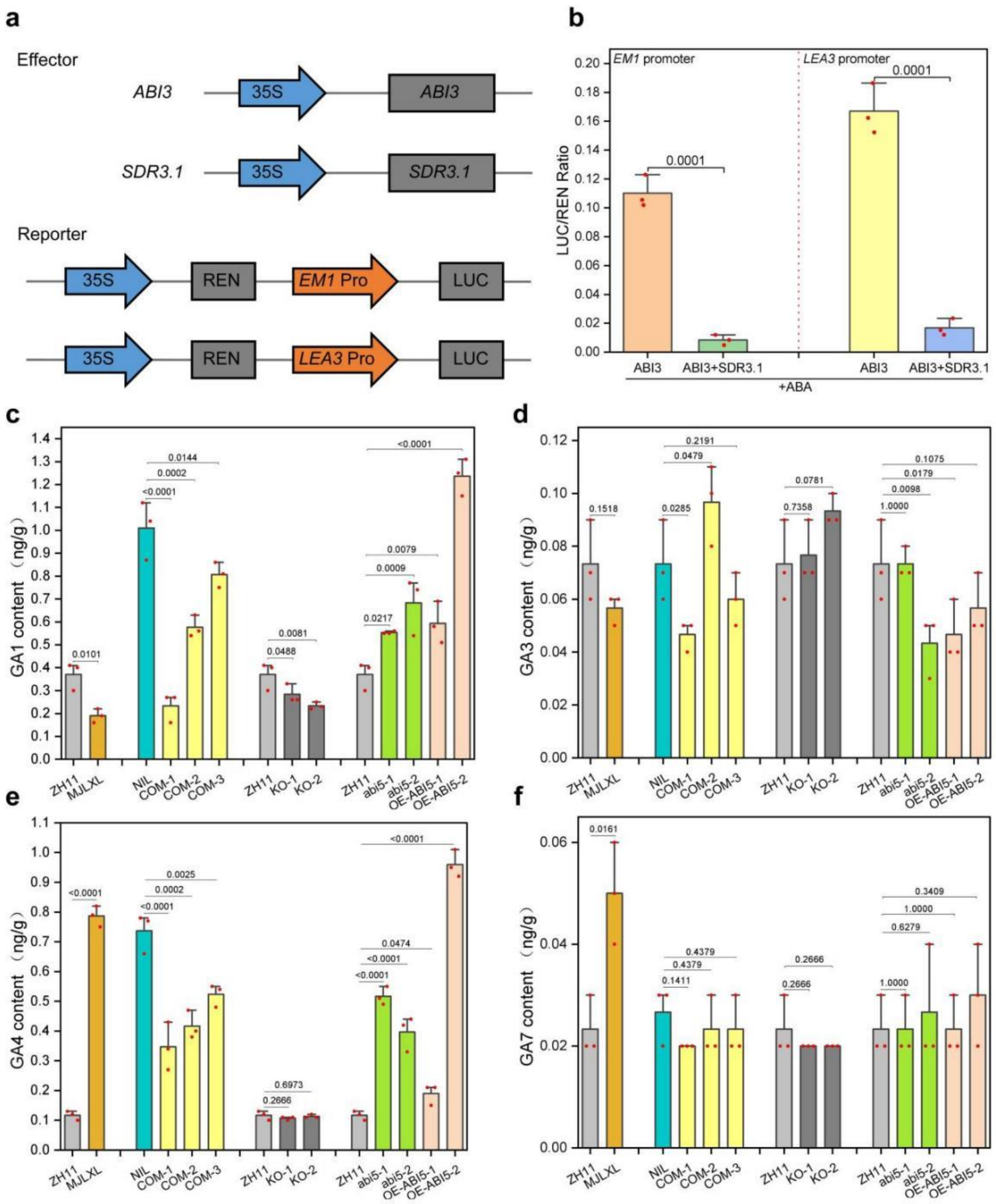

Supplementary Figure 13. ***SDR3.1* negatively regulates ABA signaling. a** Schematic of the effectors and reporter used in the transient transactivation assays. **b** *SDR3.1* repressed *ABI3* to activate *EM1* and *LEA3* in response to 5 μM ABA. REN, Renilla LUC. **c-f** GA contents of ZH11 and MJLXL; among NIL and three COM lines; among ZH11 and two *SDR3.1* knockout lines; and among ZH11, two *ABI5* knockout lines and two OE lines. Data are presented as the mean ± SD, and *P* values are indicated by two-tailed Student's *t* test. n = 3 independent experiments. Source data are provided as a Source Data file.

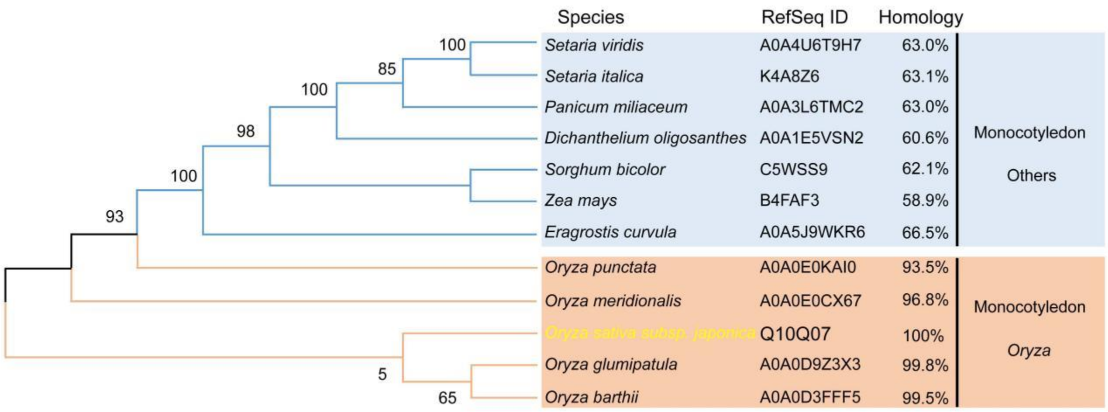

Supplementary Figure 14. Phylogenetic analysis of the SDR3.1 protein and corresponding homologous proteins.

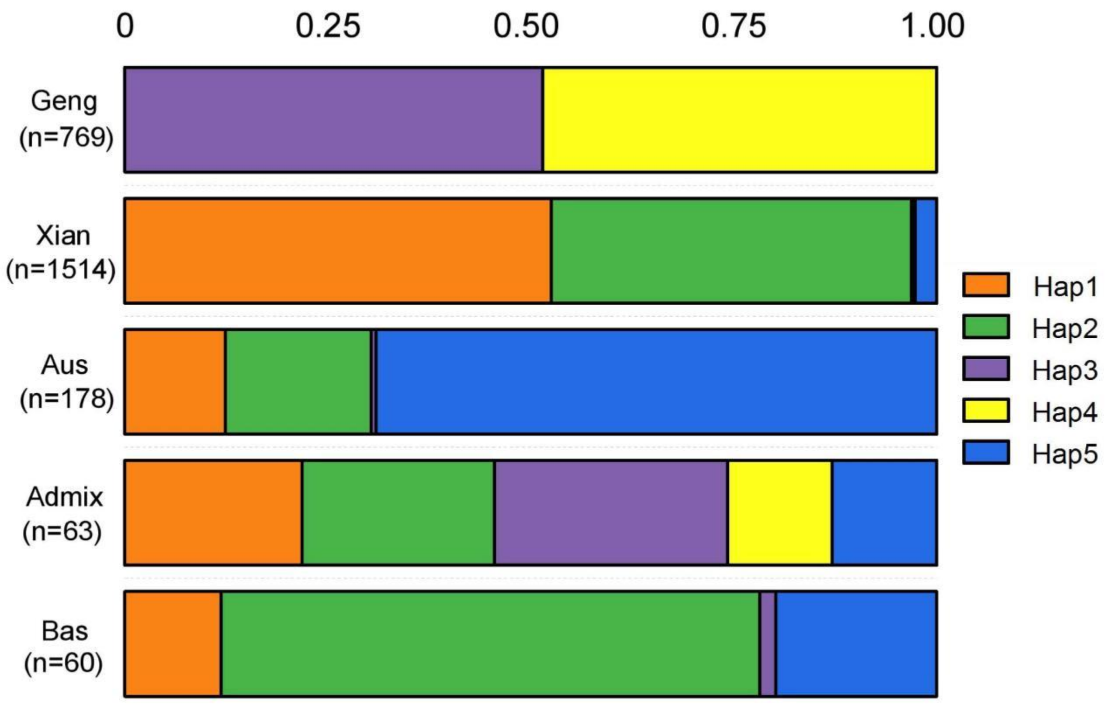

Supplementary Figure 15. Haplotype frequency of *SDR3.1* in subpopulations of 3K RG.

Source data are provided as a Source Data file.

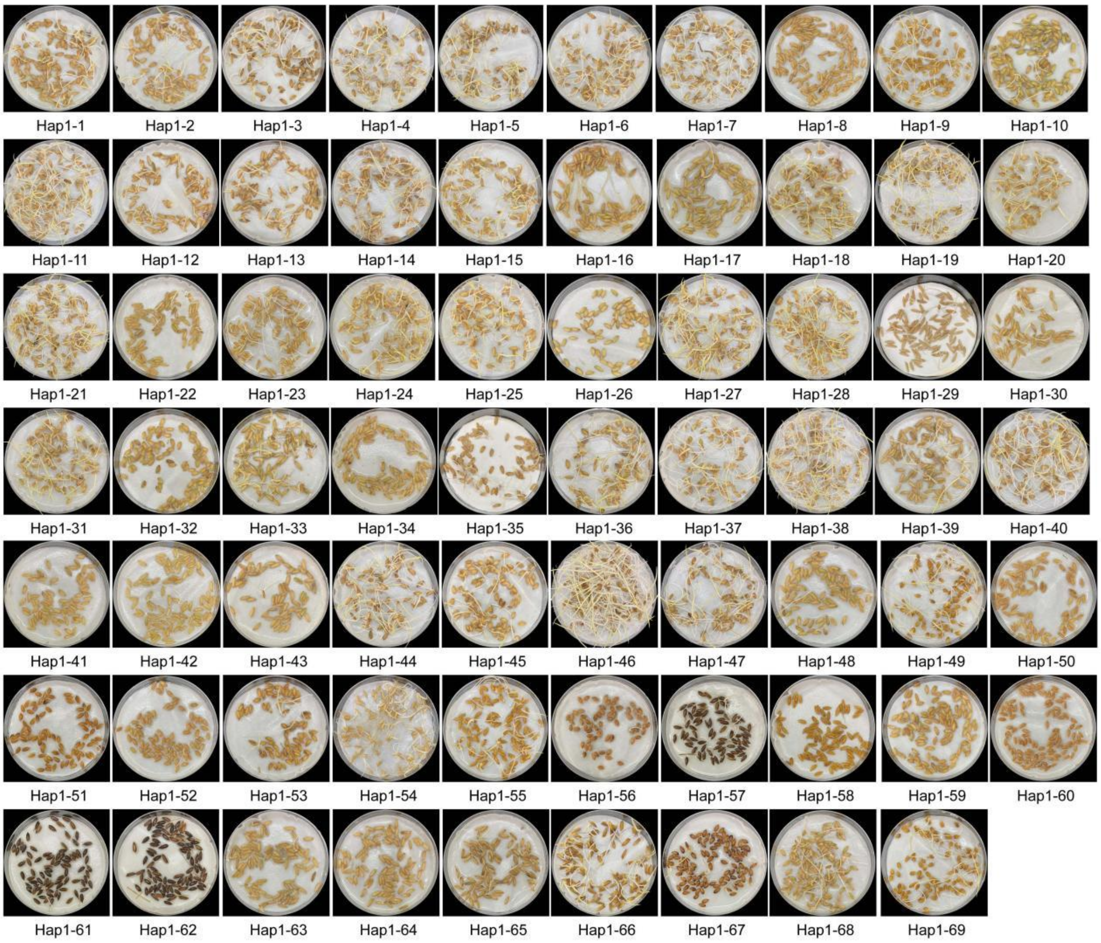

Supplementary Figure 16. The 7-day germination phenotypes of Hap1 accessions. Source data are provided as a Source Data file.

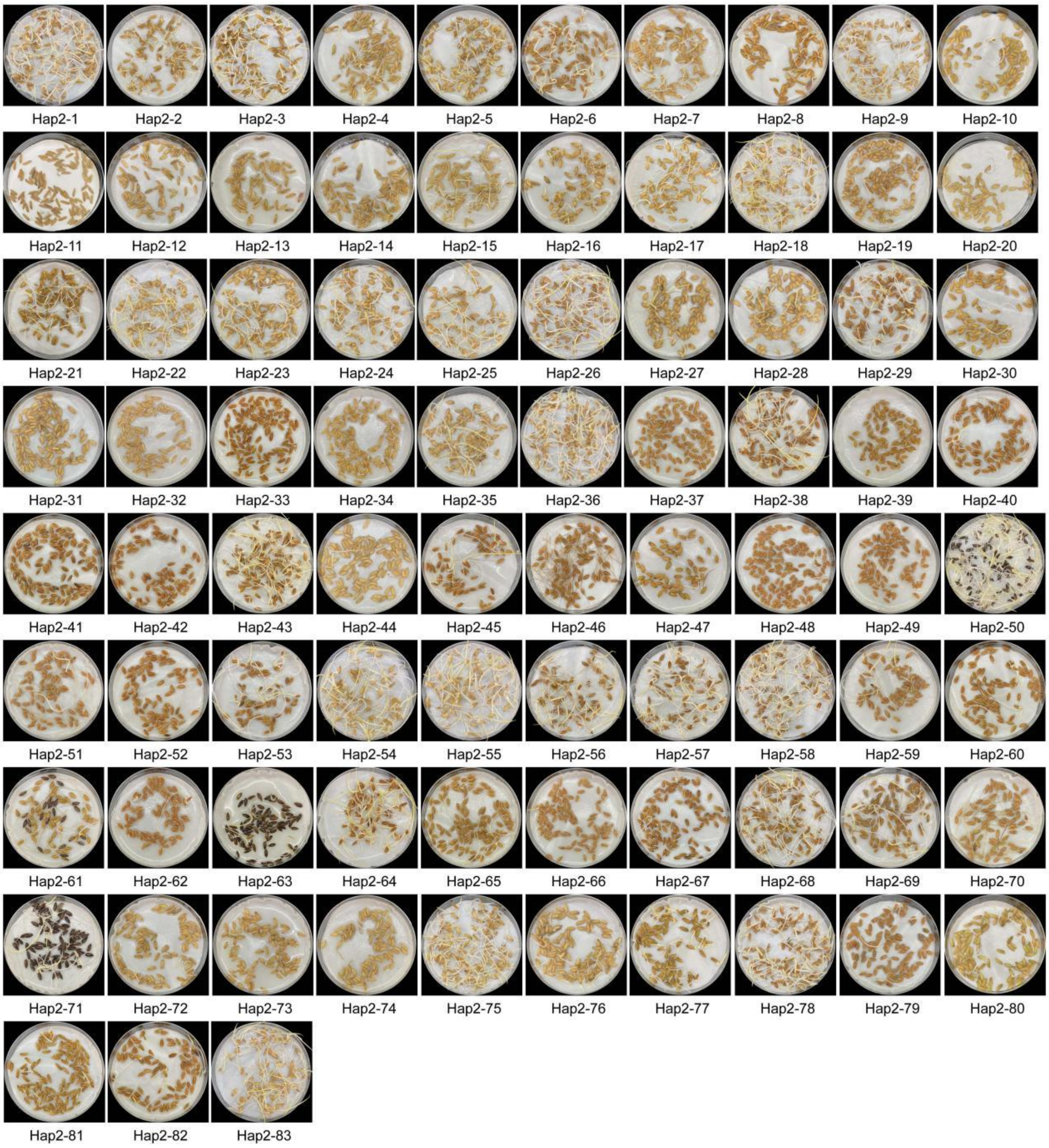

Supplementary Figure 17. The 7-day germination phenotypes of Hap2 accessions. Source data are provided as a Source Data file.

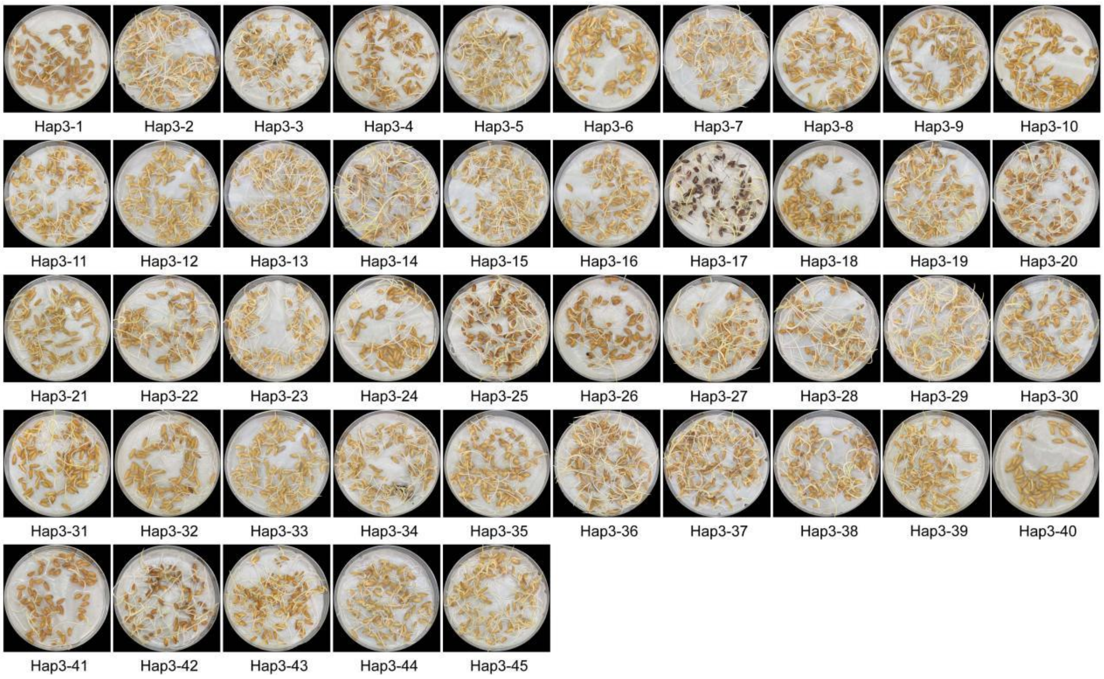

Supplementary Figure 18. The 7-day germination phenotypes of Hap3 accessions. Source data are provided as a Source Data file.

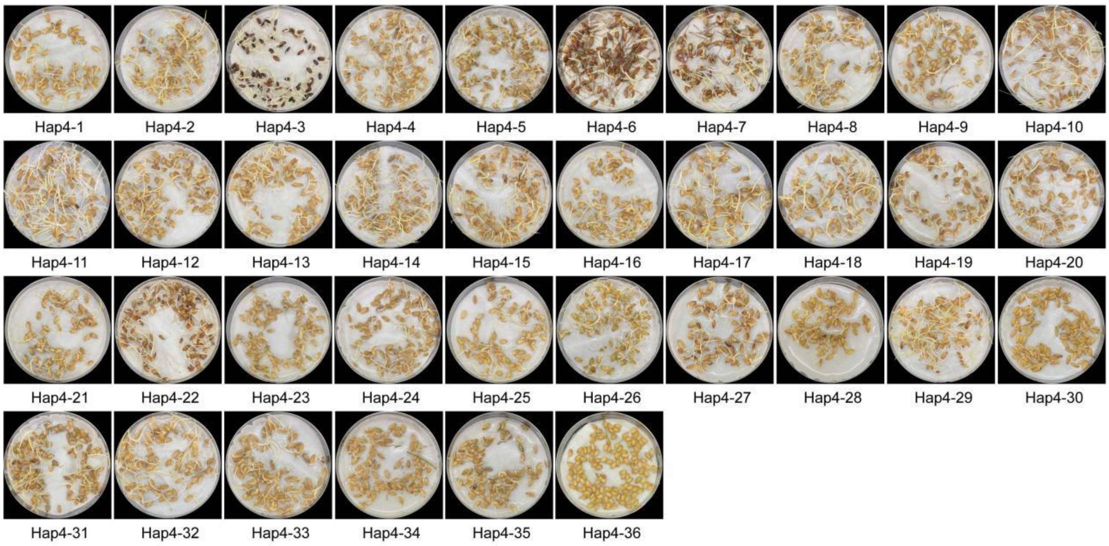

Supplementary Figure 19. The 7-day germination phenotypes of Hap4 accessions. Source data are provided as a Source Data file.

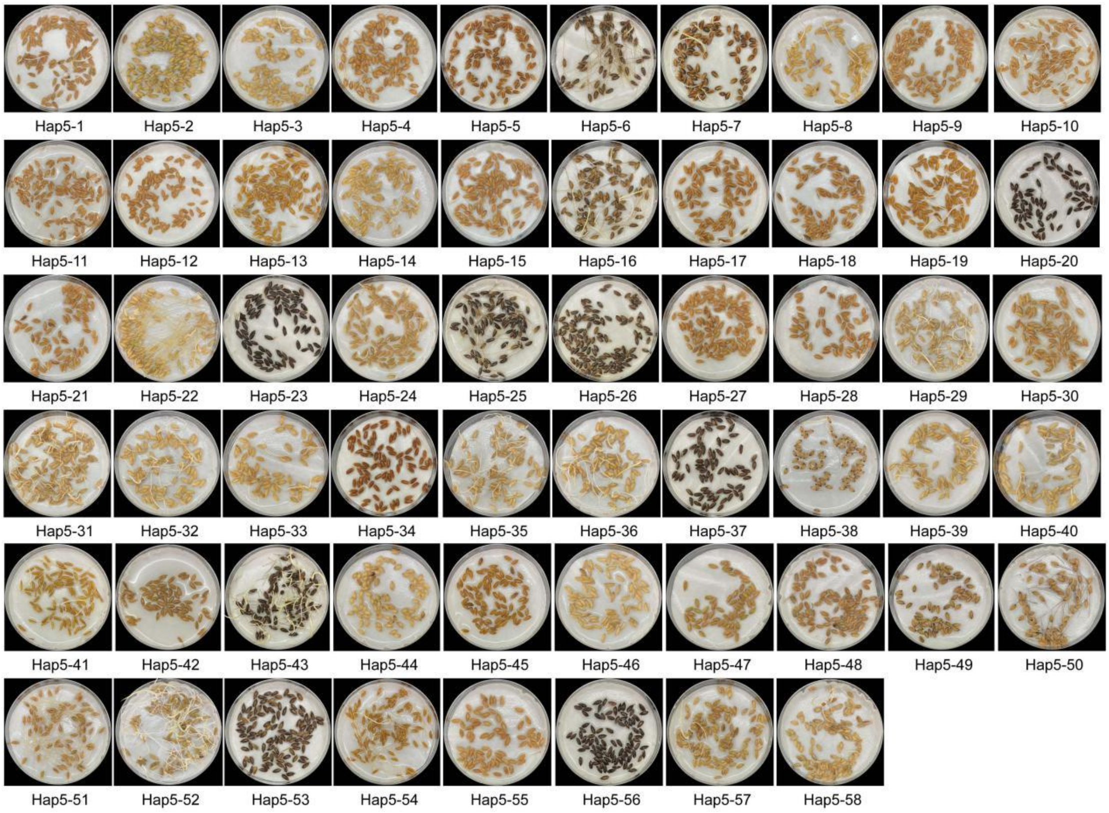

Supplementary Figure 20. The 7-day germination phenotypes of Hap5 accessions. Source data are provided as a Source Data file.

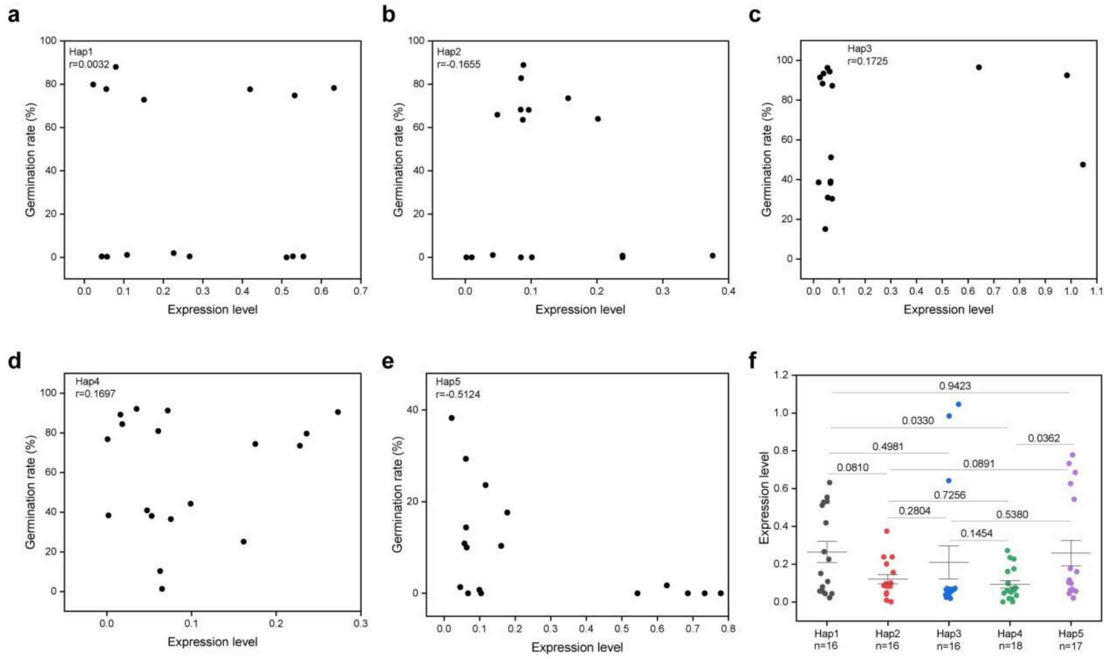

Supplementary Figure 21. Relationship between *SDR3.1* expression and dormancy. a *SDR3.1* expression levels of 16 varieties randomly selected from the Hap1 accessions. **b** *SDR3.1* expression levels of 16 varieties randomly selected from the Hap2 accessions. **c** *SDR3.1* expression levels of 16 varieties randomly selected from the Hap3 accessions. **d** *SDR3.1* expression levels of 18 varieties randomly selected from the Hap4 accessions. **e** *SDR3.1* expression levels of 17 varieties randomly selected from the Hap5 accessions. **f** *SDR3.1* expression levels with the five haplotypes. The "r" is the correlation coefficient between the germination rate and the *SDR3.1* expression level. Data are presented as the mean ± SD, and *P* values are indicated by two-tailed Student's *t* test. n = 3 independent experiments. Source data are provided as a Source Data file.

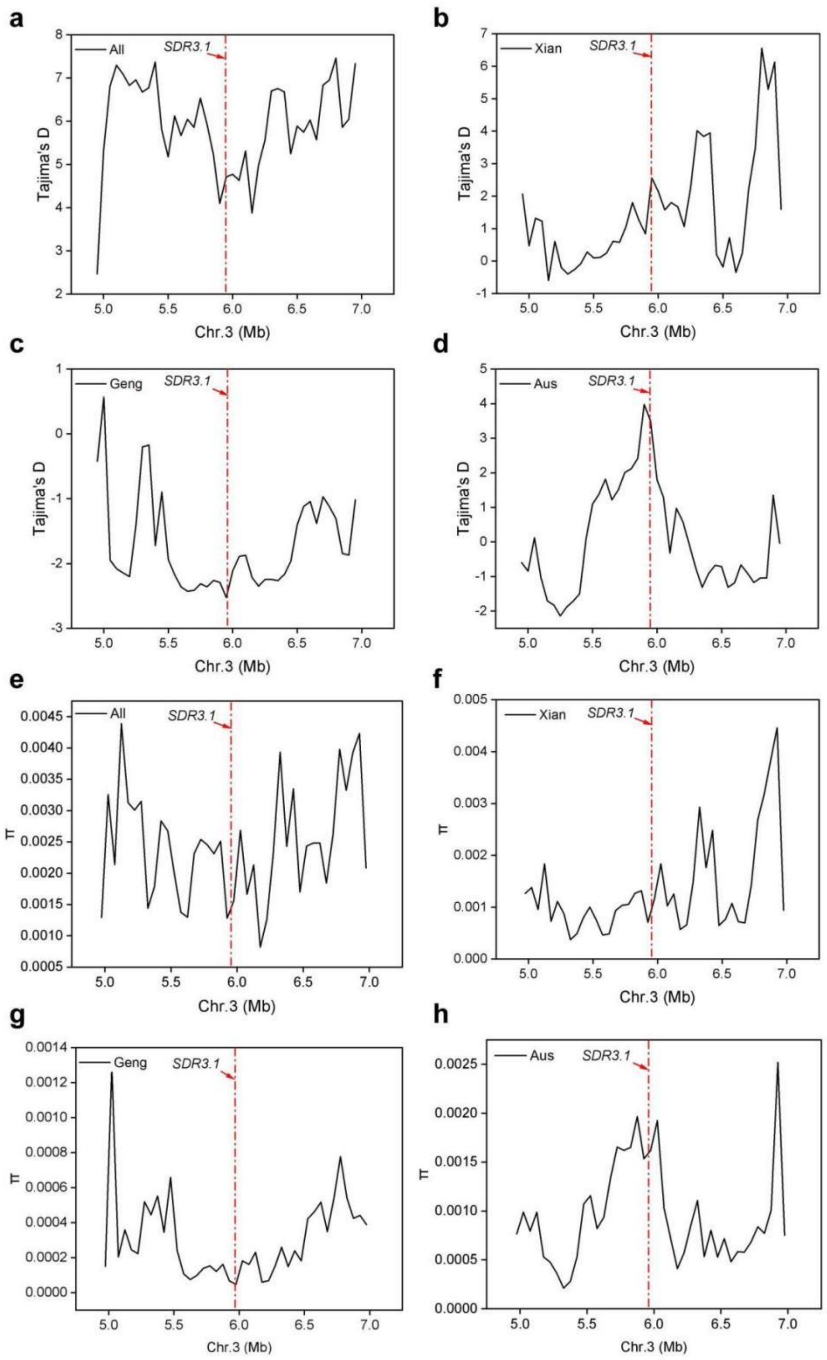

Supplementary Figure 22. Population genetic analysis of *SDR3.1*. Tajima's D for the 2-Mb genomic region flanking *SDR3.1* of All (**a**), *Xian* (**b**), *Geng* (**c**), and *Aus* (**d**) in the 3K RG. Nucleotide diversity (π) of the 2-Mb genomic region flanking *SDR3.1* of All (**e**), *Xian* (**f**), *Geng* (**g**), and *Aus* (**h**) in the 3K RG. *SDR3.1* gene is indicated by a red dashed line. Source data are provided as a Source Data file.

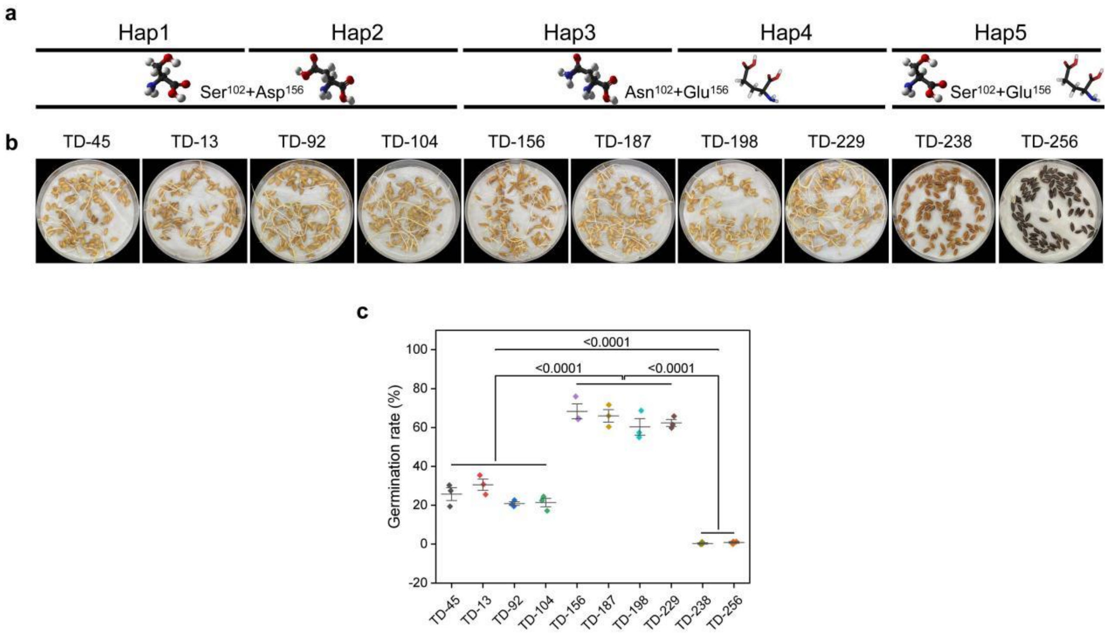

Supplementary Figure 23. Germination rate of varieties with different amino acid combinations. a Amino acid composition of five haplotypes. **b** The 7-day germination phenotype for each haplotype. **c** The 7-day germination rate for each haplotype. Data are presented as the mean ± SD, and *P* values are indicated by two-tailed Student's *t* test. n = 3 independent experiments. Source data are provided as a Source Data file.

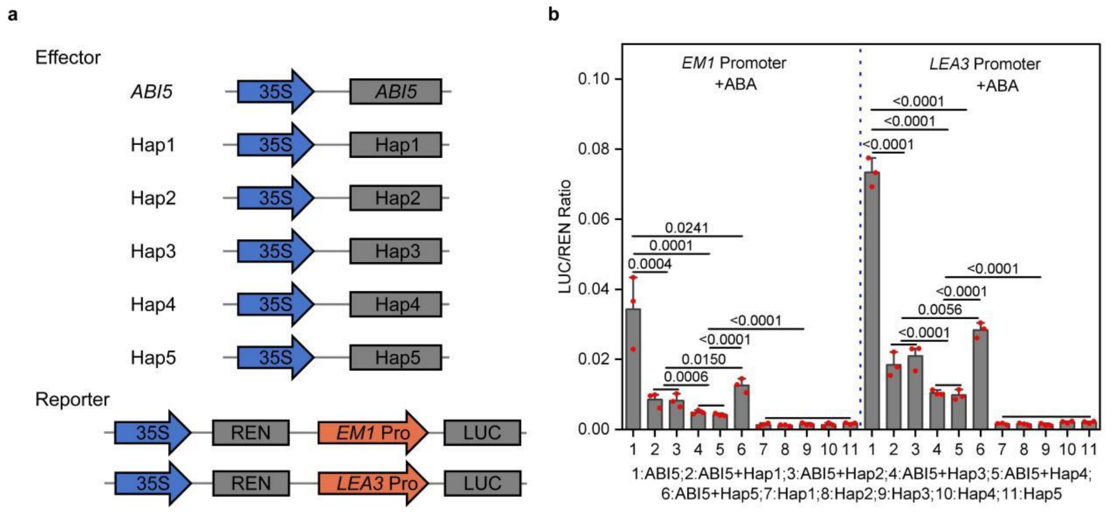

Supplementary Figure 24. Differences in the inhibitory effect of five haplotypes of SDR3.1 on ABI5. a Schematic of the effectors and reporter used in the transient transactivation assays. **b** Different SDR3.1 alleles repress ABI5 to activate *EM1* and *LEA3* in response to 5 μM ABA. REN, Renilla LUC. Data are presented as the mean ± SD, and *P* values are indicated by two-tailed Student's *t* test. n = 3 independent experiments. Source data are provided as a Source Data file.

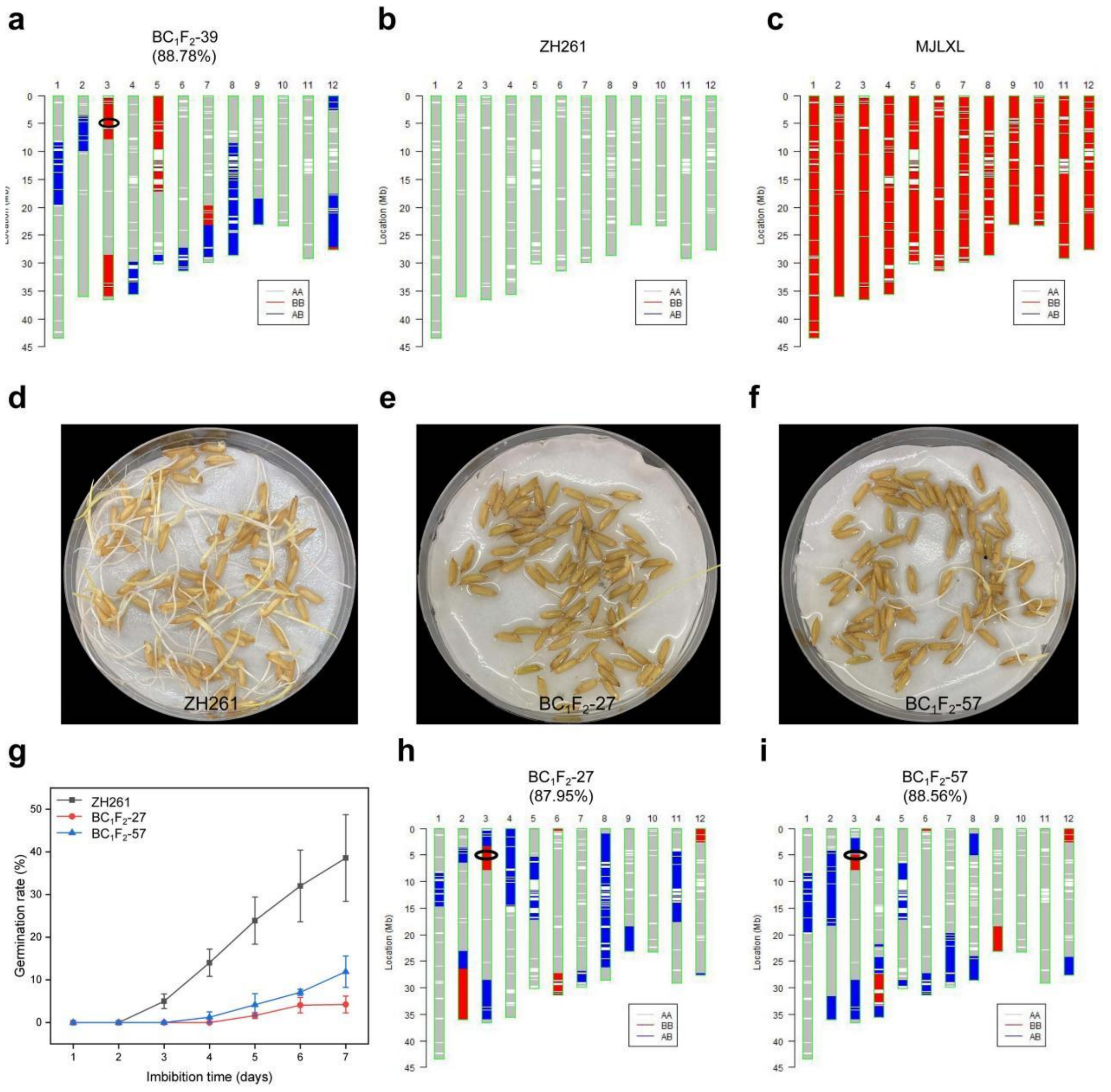

Supplementary Figure 25. The genetic background similarity and germination rate of BC_1F_2. a The genetic background similarity of BC_1F_2-39. **b** The genotype of ZH261. **c** The genotype of MJLXL. **d** The 7-day germination phenotypes of ZH261 35 DAH. **e** The 7-day germination phenotypes of BC_1F_2-27 35 DAH. **f** The 7-day germination phenotypes of BC_1F_2-57 35 DAH. **g** Time-course germination percentage of ZH261, BC_1F_2-27 and BC_1F_2-57 35 DAH. **h** The genetic background similarity of BC_1F_2-27. **i** The genetic background similarity of BC_1F_2-57. The black circle indicates the location of *SDR3.1*. Data are presented as the mean ± SD. n = 3 independent experiments. Source data are provided as a Source Data file.

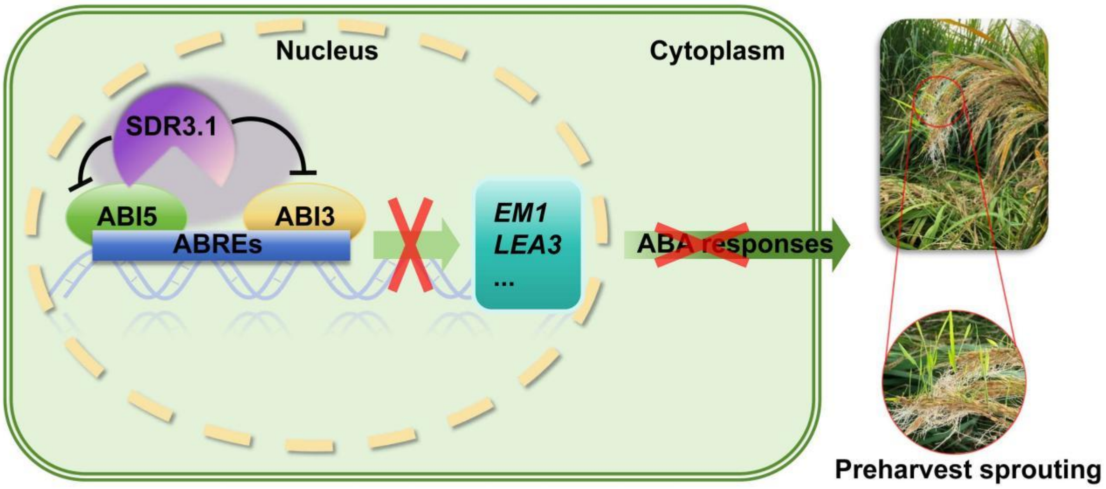

Supplementary Figure 26. Proposed working model *SDR3.1* in rice. SDR3.1 protein inhibits the transcriptional activation of ABI5 and ABI3, thereby inhibiting the expression of downstream genes such as *EM1* and *LEA3*, interrupting the ABA signaling response process, and ultimately leading to seed germination.

Supplementary Table 1. List of QTL parameters.

Population	Trait	QTL	Chr.	Interval	LOD	Additive	Dominant	PVE(%)
		qSDR2.1	2	2-39-2-43	2.89	-0.06	-0.06	6.25
BC_3F_2	Germination rate	*qSDR3.1*	3	3-15-3-21	11.75	-0.16	-0.02	26.38
		qSDR3.2	3	3-27-3-33	5.67	-0.10	0.00	9.91

Supplementary Table 2. List of candidate genes within the localization interval.

Gene ID	Annotation
LOC_Os03g11520	Expressed protein
LOC_Os03g11530	Nucleotide pyrophosphatase/phosphodiesterase, putative, expressed
LOC_Os03g11540	RPA1B - Putative single-stranded DNA binding complex subunit 1, expressed
LOC_Os03g11550	Mediator of OsbZIP46 deactivation and degradation
