# Supplementary material for: A mediator of OsbZIP46 deactivation and degradation negatively regulates seed dormancy in rice
Source: Nat Commun. 2024 Feb 7;15:1134. doi: 10.1038/s41467-024-45402-z (PMC10850359; doi:10.1038/s41467-024-45402-z)
Supplement: Supplementary file 3 — Description of Additional Supplementary Files [file 41467_2024_45402_MOESM3_ESM.pdf]

Description of Additional Supplementary Files

File Name: Supplementary Data 1
Description: The genotyping data for the BC_3F_1 plant.

File Name: Supplementary Data 2
Description: List of primer pairs used in this study.
